# Dynamic Measurement and Structural Decomposition of Deep Poverty in Contiguous Destitute Areas

**DOI:** 10.1155/2021/9461652

**Published:** 2021-09-16

**Authors:** Da-Yang Zhang, Rui-Feng Peng, Jin-Biao Zheng, You-Qun Wu, Xiao-Yi Wang

**Affiliations:** ^1^School of Economics, Anhui University of Finance & Economics, Bengbu, Anhui 233030, China; ^2^School of Marxism, Shanghai University of Finance and Economics, Shanghai 200433, China; ^3^School of Economics & Managemengt, Anhui Agricultural University, Hefei, Anhui 230036, China

## Abstract

Based on the sample data from 2005 to 2019, this paper calculates the poverty nature of contiguous destitute areas through FGT index and its decomposition and systematically analyzes the impact of economic growth, inequality, and population change on poverty change. From the decomposition results of poverty change, we can see that, first, economic growth, inequality, and population change have different impacts on poverty change in counties and rural areas, and inequality and population mobility have widened the gap between them; second, population factor has always played a key role in the change of poverty, and the deceleration of population growth has a more significant impact on poverty change; third, the impact of the mobility on the poverty change of the counties is different from that of the rural areas. Accordingly, the paper puts forward some countermeasures and suggestions, such as promoting the organic connection between rural revitalization and poverty alleviation, speeding up rural governance, and promoting the process of urbanization.

## 1. Introduction

Deep poverty is a major economic problem in current social development. China is committed to eliminating poverty and building itself into a modern powerful country in an all-round way. With the effective combination of rural revitalization strategy and poverty alleviation, great achievements have been made in poverty management. In contiguous destitute areas, the recognition degree of population deep poverty is high, and the deep poverty status shows “dynamic, long-term, and fragile” and other characteristics and changing rules, which is an important problem in the current deep poverty governance process [1]. The study on the change of deep poverty in this area is conducive to the exploration of the influencing factors of deep poverty change and the formulation of appropriate poverty alleviation strategies for different regions and groups to effectively solve the persistent problem of deep poverty.

Estimation and decomposition of deep poverty are an important direction for the study of deep poverty. Research and design of structural decomposition for deep poverty are conducted. Decomposition of deep poverty into independent factors is conducive to the study of “why poverty” and the reasonable design of deep poverty governance plan. With the deepening of deep poverty decomposition research, the role of population factors in the change of deep poverty has become the research focus of scholars. Based on the FGT index, this study calculated the change of deep poverty of contiguous destitute areas and conducted structural decomposition. Explanation of the total effect of deep poverty change from the three directions of economic growth, inequality, and population change is conducive to the study of the change in the nature of poverty in this area, the formulation of reasonable development strategies, and the consolidation of poverty alleviation achievements.

## 2. Documentary Introduction

The identification and decomposition of deep poverty are the basis of targeted poverty alleviation. Traditional poverty analysis is based on single-dimension income poverty, which does not take the future welfare of the poverty families into consideration in the analysis based on a specific time point [2]. Based on Sen's ability poverty analysis, scholars conducted research on multidimensional poverty, showing that the causes of poverty are no longer limited to income, but also deprivation of ability, such as malnutrition, chronic epidemic disease, and illiteracy [3]. At present, domestic researches on deep poverty mostly focus on the governance of deep poverty. For example, the path analysis of deep poverty, the governance mechanism of deep poverty, and the influencing factors of deep poverty are studied [4–6].

Accurate identification of deep poverty groups is the main problem in deep poverty research, and the quality of poverty reduction policies mainly depends on the identification of deep poverty groups. With the deepening of the research on deep poverty, scholars have introduced the axiom system of deep poverty into the existing basic poverty measure to improve it. From the perspective of comprehensive welfare security, the deep poverty status of each region is measured, studied, and compared comprehensively [7]. Some scholars also calculated the degree of rural deep poverty from the perspective of digital inclusive finance based on the constraint conditions of information gap [8]. Some other scholars calculated deep poverty based on the vulnerability axiom, and while meeting the prospective requirements, they could also deeply analyze the governance effect of deep poverty [9].

In terms of the method of poverty decomposition, there are mainly two types. The first type is based on A-F method. For example, the deep poverty dimension is introduced to construct a multidimensional deep poverty index with subgroup decomposition, and the double-critical value (A-F) method is used to study deep poverty decomposition [10, 11]. The second category is the variable decomposition of FGT poverty index. For example, FGT index is used to determine the deterioration of poverty. FGT index decomposition is divided into intragroup and intergroup effects for poverty decomposition study [12, 13]. There are few studies on the measurement of deep poverty of contiguous destitute areas and the change of their nature. Only the measurement of poverty in the Ta-pieh Mountains is available. Measuring the degree of poverty and decomposition in the whole contiguous destitute areas is even less common [14].

To sum up, scholars have achieved a lot of research results in the measurement and decomposition of deep poverty, but there are the following shortcomings: first, when measuring the change of deep poverty based on the economic growth and inequality of the whole country, regional differences are ignored; second, there are high requirements for sample data, most of which are based on static cross-section data or intertemporal data. The covered years of deep poverty are too short, and the continuity of deep poverty time cannot be reflected; third, few scholars consider population change in deep poverty change. Such practice is flawed because population change will dilute the effect of total income and economic growth, and population change itself has the effect of poverty reduction [15]. Based on this, this paper tries to make efforts from the following aspects: first, the current discussion on strategies, paths, and countermeasures of the degree of poverty of contiguous destitute areas is fully absorbed, and the deep poverty index is used to carry out a measurement analysis of the degree of deep poverty in this area [16]; second, time series data are introduced to ensure the time continuity of deep poverty; third, the population change is decomposed from the deep poverty index to observe the impact of population change on the change of deep poverty.

## 3. Methods and Modeling

### 3.1. Constructing Deep Poverty Index

FGT index has been widely used in the study and measurement of deep poverty and has good decomposition and addition. However, it is not sensitive to the distribution and deprivation of deep poverty during the deep poverty addition. For this reason, scholars have carried out a lot of improvement studies, formula shown as follows:

(1) $$\begin{array}{c} P_{\alpha t}={\sum_{it=1}^{nt}b_{i_{t}}\left(\frac{z-x_{it}}{z}\right)}^{\alpha };\;\alpha \geq 0. \end{array}$$

And in chronological order, there is *t*=1,2,…, *n*, *it*=1*t*, 2*t*,…, *nt* in each time period, *x*_*it*_ represents a unit that registers in the nondecreasing order, *z* represents deep poverty line, *b*_*it*_ represents ratio of population in the register of deep poverty, and *α* ≥ 0,  *α*=0,1,2 correspondingly represent the difference between the deep poverty and the deep poverty created by the square of the deep poverty gap. If the selection time is long, the deep poverty line of the same period will be substituted into the calculation when the deep poverty line is introduced, and the influence of price changes will be ignored, which will inevitably enlarge the poverty gap. Therefore, *z*_*t*_ is considered as the deep poverty line. According to the designated time period, the corresponding deep poverty line is introduced, formula shown as follows:

(2) $$\begin{array}{c} P_{\alpha t}={\sum_{it=1}^{nt}b_{it}\left(\frac{z_{t}-x_{it}}{z_{t}}\right)}^{\alpha };\;\alpha \geq 0. \end{array}$$

### 3.2. Decomposition of Deep Poverty Index

In order to facilitate the decomposition of all factors in the deep poverty index, the deep poverty factor index is constructed. In addition, the idea of deep poverty index decomposition proposed by Mishra [17] is used for reference, shown as follows:

(3) $$\begin{array}{c} P_{\alpha t|\gamma \tau }={\sum_{it=1}^{nt}b_{it}\left(\frac{z_{t}-\lambda_{\nu }x_{it}}{z_{t}}\right)}^{\alpha };\;\alpha \geq 0,\;\gamma_{\tau }=m_{\tau },X_{\tau },n_{\tau },\\ \lambda \nu =\left(\frac{m_{\tau }}{m_{t}}\right),\left(\frac{X_{\tau }}{X_{t}}\right),\left(\frac{n_{\tau }}{n_{t}}\right);\;\nu =m,X,n. \end{array}$$

Multiplying each deep poverty unit by a constant *λ*_*ν*_, *λ*_*m*_, *λ*_*X*_, *λ*_*n*_ correspondingly represent the mean income, total income, and the ratio of total population in these two time periods.

As for the decomposition of FGT index, scholars generally classify it as economic growth and inequality effect. The growth effect is based on the change of average income, and the rest is classified as inequality effect. The change of population is hidden in the change of average income and ignored. If the growth effect is extracted from the total income, the formula can be decomposed as

(4) $$\begin{array}{c} \Delta P_{\alpha }=\frac{1}{2}\left\{\left(P_{\alpha 1|x2}-P_{a1}\right)+P_{\alpha 2}-P_{\alpha 2|x1}\right\}+\frac{1}{2}\left\{\left(P_{\alpha 2}-P_{\alpha 1|x2}+P_{\alpha 2|x1}-P_{\alpha 1}\right)\right\}. \end{array}$$

If there is no change in population in two periods, then the economic growth effects obtained based on the change of average income and total income are equal, which means *n*_*τ*_/*n*_*t*_=*X*_*τ*_/*X*_*t*_. All incomes of all populations change in the same proportion during the period of investigation, which is impossible to happen. Therefore, changes in population are hidden in changes in average incomes and are not separated out. In practice *n*_*τ*_/*n*_*t*_ ≠ *X*_*τ*_/*X*_*t*_, the situation that the economic growth effects obtained based on the change of average income and total income are equal does not exist, and population needs to be decomposed in deep poverty. Then we have the following hypothesis.

**Hypothesis 1 .**

Population change is one of the factors affecting the change of poverty.

In formula (4), the growth effect can be divided into the average income effect and the additional effect of total income. As a result,

(5) $$\begin{array}{c} \Delta P_{\alpha }=\frac{1}{2}\left\{\left(P_{\alpha 1|m2}-P_{\alpha 1}\right)+\left(P_{\alpha 2}-P_{\alpha 2|m1}\right)\right\}+\frac{1}{2}\left\{\left(P_{\alpha 1|x2}-P_{\alpha 1|m2}\right)+\left(P_{\alpha 2|m1}-P_{\alpha 2|x1}\right)\right\}+\frac{1}{2}\left\{\left(P_{\alpha 2}-P_{\alpha 1|x2}\right)+\left(P_{\alpha 2|x1}-P_{\alpha 1}\right)\right\}. \end{array}$$

These three parts are the growth effect of average income, the growth effect after deducting the impact of average income on total income, and inequality effect, respectively. However, inequality effect includes inequality influence and the influence of total population change, and the influence of total population change is extracted from the inequality effect, which can be decomposed as

(6) $$\begin{array}{c} \Delta P_{\alpha }=\frac{1}{2}\left\{\left(P_{\alpha 1|m2}-P_{\alpha 1}\right)+\left(P_{\alpha 2}-P_{\alpha 2|m1}\right)\right\}+\frac{1}{2}\left\{\left(P_{\alpha 1|x2}-P_{\alpha 1|m2}\right)+\left(P_{\alpha 2|m1}-P_{\alpha 2|x1}\right)\right\}\\ +\frac{1}{2}\left\{\left(P_{\alpha 2|n1}-P_{\alpha 1|x2}\right)+\left(P_{\alpha 2|x1}-P_{\alpha 1|n2}\right)\right\}+\frac{1}{2}\left\{\left(P_{\alpha 2}-P_{\alpha 2|n1}\right)+\left(P_{\alpha 1|n2}-P_{\alpha 1}\right)\right\}. \end{array}$$

The four parts mentioned above are, respectively, the growth effect of average income, the growth effect after deducting the impact of average income on total income, the inequality effect, and the influence of the change of total population. When the population of two periods is consistent, the above four parts can be combined into growth effect and inequality.

In formula (6), the four parts are summarized as growth effect, inequality, and population change effect. The result of decomposition depends on the selection of calculation sequence and base period. Three effects are calculated in three sequences. There are six ways of decomposition, shown as follows:

(i) Economic growth, inequality, and population:
  (7) $$\begin{array}{c} \Delta P_{\alpha }=\left(P_{\alpha 1|x2}-P_{\alpha 1}\right)+\left(P_{\alpha 2|n1}-P_{\alpha 1|x2}\right)+\left(P_{\alpha 2}-P_{\alpha 2|n1}\right). \end{array}$$
(ii) Economic growth, population, and inequality:
  (8) $$\begin{array}{c} \Delta P_{\alpha }=\left(P_{\alpha 1|x2}-P_{\alpha 1}\right)+\left(P_{\alpha 2|l1}-P_{\alpha 1|x2}\right)+\left(P_{\alpha 2}-P_{\alpha 2|l1}\right). \end{array}$$
(iii) Inequality, economic growth, and population:
  (9) $$\begin{array}{c} \Delta P_{\alpha }=\left(P_{\alpha 1|l2}-P_{\alpha 1}\right)+\left(P_{\alpha 2|n1}-P_{\alpha 1|l2}\right)+\left(P_{\alpha 2}-P_{\alpha 2|n1}\right). \end{array}$$
(iv) Inequality, population, and economic growth:
  (10) $$\begin{array}{c} \Delta P_{\alpha }=\left(P_{\alpha 1|l2}-P_{\alpha 1}\right)+\left(P_{\alpha 2|x1}-P_{\alpha 1|l2}\right)+\left(P_{\alpha 2}-P_{\alpha 2|x1}\right). \end{array}$$
(v) Population, economic growth, and inequality:
  (11) $$\begin{array}{c} \Delta P_{\alpha }=\left(P_{\alpha 1|n2}-P_{\alpha 1}\right)+\left(P_{\alpha 2|l1}-P_{\alpha 1|n2}\right)+\left(P_{\alpha 2}-P_{\alpha 2|l1}\right). \end{array}$$
(vi) Population, inequality, and economic growth:
  (12) $$\begin{array}{c} \Delta P_{\alpha }=\left(P_{\alpha 1|n2}-P_{\alpha 1}\right)+\left(P_{\alpha 2|x1}-P_{\alpha 1|n2}\right)+\left(P_{\alpha 2}-P_{\alpha 2|x1}\right). \end{array}$$

In the above six decomposition equations, each of which contains three components, taking economic growth, inequality, and population decomposition for example, (*P*_*α*1*|x*2_ − *P*_*α*1_) is used to explain the economic growth effect, where population and inequality are fixed in the base period. (*P*_*α*2*|n*1_ − *P*_*α*1*|x*2_) is used to explain inequality effect, in which population is fixed in the base period and total income is fixed in the reporting period. (*P*_*α*2_ − *P*_*α*2*|n*1_) is used to explain population change, among which economic growth and inequality are both fixed in the reporting period, and population is fixed in the base period. When the base period changes, the order of the three components is reversed. In this equation, population is a direct influence factor, and the inequality effect is affected by the change of population, while the economic growth effect is affected by the change of population. The three parts of economic growth effect, inequality effect, and population change are included in the six decomposition equations. Therefore, when calculating the contribution of single effect to poverty index,

(13) $$\begin{array}{c} \Delta P_{\alpha j}=\frac{1}{S}\sum_{s}\Delta P_{\alpha js}=;\;j=X,L,n;\;s=1,\ldots ,s,\\ \Delta P_{\alpha x}=\frac{1}{3}\left(P_{\alpha 1|x2}-P_{\alpha 1}\right)+\frac{1}{6}\left(P_{\alpha 2|n1}-P_{\alpha 1|l2}\right)+\frac{1}{6}\left(P_{\alpha 2|l1}-P_{\alpha 1|n2}\right)+\frac{1}{3}\left(P_{\alpha 2}-P_{\alpha 2|x1}\right),\\ \Delta P_{al}=\frac{1}{3}\left(P_{\alpha 1|l2}-P_{\alpha 1}\right)+\frac{1}{6}\left(P_{\alpha 2|x1}-P_{\alpha 1|n2}\right)+\frac{1}{6}\left(P_{\alpha 2|n1}-P_{\alpha 1|x2}\right)+\frac{1}{3}\left(P_{\alpha 2}-P_{\alpha 2|l1}\right),\\ \Delta P_{\alpha n}=\frac{1}{3}\left(P_{\alpha 1|n2}-P_{\alpha 1}\right)+\frac{1}{6}\left(P_{\alpha 2|l1}-P_{\alpha 1|x2}\right)+\frac{1}{6}\left(P_{\alpha 2|x1}-P_{\alpha 1|l2}\right)+\frac{1}{3}\left(P_{\alpha 2}-P_{\alpha 2|n1}\right). \end{array}$$

**Hypothesis 2 .**

Economic growth, inequality, and population change are all key factors affecting the change of poverty, and they are independent of each other.

The change of poverty can be obtained from the sum of the above three effects:

(14) $$\begin{array}{c} \Delta P_{\alpha }=\sum_{j}\Delta P_{\alpha j};\;j=X,L,n. \end{array}$$

### 3.3. Decomposition of Intragroup and Intergroup Effect

Deep poverty index changes are caused by economic growth effect, inequality, and population effect. These three components all contain intragroup effect and intergroup effect, so they can be subgroup decomposed, and formula (14) is deformed into [18]

(15) $$\begin{array}{c} \Delta P_{\alpha }=\sum_{k=1}^{K}\Delta P_{\alpha k}=\sum_{j}\sum_{k=1}^{K}\Delta P_{\alpha jk};\;j=X,L,n,\\ \Delta P_{a}=\left(P_{2}-P_{1}\right)=\left(\sum_{k=1}^{K}b_{k2}\Delta P_{\alpha k2}\right)-\left(\sum_{k=1}^{K}b_{k1}\Delta P_{\alpha k1}\right)\\ =\frac{\left(\left(\sum_{k=1}^{K}b_{k2}\Delta P_{\alpha k}\right)+\left(\sum_{k=1}^{K}b_{k1}\Delta P_{ak}\right)+\left(\sum_{k=1}^{K}P_{\alpha k2}\Delta b_{k}\right)+\left(\sum_{k=1}^{K}P_{\alpha k1}\Delta b_{k}\right)\right)}{2}\\ =\left(\left(\sum_{k=1}^{K}\frac{\left(b_{k2}+b_{k1}\right)}{2}\right)\Delta P_{ak}\right)+\left(\left(\sum_{k=1}^{K}\frac{\left(P_{ak2}+P_{ak1}\right)}{2}\right)\Delta b_{k}\right). \end{array}$$

This means that decomposition deep poverty change indicated in equation (13) could be further decomposed to within-group and between-group effects, where the intragroup effect can be decomposed into the influence of economic growth, inequality, and population effect on the change of population share, which is independent from the population share in the intergroup effect and other influence effects, namely,

(16) $$\begin{array}{c} \Delta P_{\alpha }=\left(\left(\sum_{j}\sum_{k=1}^{K}\frac{\left(b_{k2}+b_{k1}\right)}{2}\Delta P_{\alpha jk}\right)+\left(\sum_{k=1}^{K}\frac{\left(P_{\alpha k2}+P_{\alpha k1}\right)}{2}\Delta b_{k}\right)\right). \end{array}$$

In deep poverty index decomposition, both intragroup effect and intergroup effect contain components related to population. One is one of the subcomponents of intragroup effect, and the other is the change of population share in intergroup effect. The increase of total population while the income and inequality remain constant will aggravate the deep poverty effect. Changes in population shares within groups, such as those between urban and rural areas, will inevitably have beneficial or adverse effects on one of the groups.

### 3.4. Source and Processing of Data

The data period used in this paper is from 2005 to 2019. In view of the availability of data, 11 concentrated contiguous destitute areas are taken as samples. They include Liu-p'an Mountains, Tsinling Mountains, Wuling Mountains, Wumeng Mountains, Yunnan, Guizhou and Guangxi rocky desertification areas, western Yunnan border mountainous areas, Greater Khingan Range, Yanshan Mountains, T'ai-hang Mountains, Lvliang Mountains, Ta-pieh Mountains, Luoxiao Mountains, and other regions. The data required are from China County Statistical Yearbook, Poverty Monitoring Report of Rural China, and the statistical yearbooks and bulletin of corresponding cities and counties, etc., and the mean difference interpolation method is adopted for some missing values.

Poverty line refers to the minimum cost of goods and services necessary for basic survival under a certain period of time, space, and social development stage. Since the establishment of the poverty line in 1985, the poverty line has been adjusted year by year according to the price index. In order to reduce the amplification of poverty by using a single poverty line, the 2005–2019 historical poverty lines are introduced into the calculation. Contiguous poverty-stricken areas are directly affiliated to deep poverty areas in the first stage of poverty alleviation, and the comparison of absolute poverty line and deep poverty line should be introduced to study the nature of poverty in this area [19]. The definition of deep poverty criteria has been controversial among scholars at home and abroad. Some scholars adopted the mean income as the standard for deep poverty, but the poverty gap would lead to the failure or overidentification of some deep poverty groups. In addition, the mobility between urban and rural areas is enhanced, and relative low-income groups widely exist in cities, so 1 deep poverty standard alone cannot effectively identify complete deep poverty groups. For this reason, the method of income ratio is adopted to measure deep poverty, and the standard lines of deep poverty are set, respectively, on the basis of the median per capital disposable income of urban residents of the current period, the median per capital disposable income of national residents of 50%, and the median per capital disposable income of rural residents of 60%.

## 4. Multidimensional Dynamic Measurement of Poverty

The population share of poverty unit, income, and the poverty line of previous years are introduced into the FGT index to calculate the poverty situation of contiguous destitute areas from 2005 to 2019. The results are shown in Table 1.

According to the poverty index, absolute poverty in contiguous destitute areas has been alleviated in 2011, and most of the poverty households have quitted absolute poverty. The corresponding poverty alleviation stage is extensive poverty alleviation. Under the promotion of the poverty alleviation policy of flood irrigation, most of the poverty population in the poverty counties in this area has been out of absolute poverty, and the poverty groups are becoming more and more cohesive as the concentration of poverty groups becomes more and more dispersed. From 2014 to 2019, the change of the poverty index was relatively gentle. Under the promotion of the targeted poverty alleviation policy, the absolute poverty level decreased step by step, while the remaining poverty population decreased step by step. According to the deep poverty measurement index, the poverty population in contiguous destitute areas that has dropped out of absolute poverty will fall into deep poverty after being lifted out of poverty. Taking 60% of the median per capital income of rural residents and 50% of the national median per capital income as the standard, the calculation results show that the degree of deep poverty of contiguous destitute areas has been alleviated. However, a small proportion of the population is still in deep poverty. Taking 40% of the median per capital income of urban residents as the standard, the calculation results show that, in contiguous destitute areas, although the degree of deep poverty has been alleviated to some extent, there is still a part of population in deep poverty.

The governance nature of poverty in contiguous destitute areas has changed from absolute poverty elimination to deep poverty alleviation. Since the implementation of targeted poverty alleviation in 2014, the poverty index has been continuously optimized, and scattered units of absolute poverty have quitted absolute poverty one after another. However, it can be seen from the measurement results of deep poverty index that contiguous destitute areas are in deep poverty, indicating that the governance direction of this area should be changed to deep poverty governance. The results based on the per capital income of rural residents and the per capital income of national residents show that the degree of deep poverty in this area is reduced, while the results based on the per capital income of urban residents show that the degree of deep poverty in this area is relatively deep, indicating that although this area is basically out of absolute poverty, there is still a gap between the economic development and the national average level. Some people who are out of deep poverty in this area will still be identified as deep poverty population in the towns.

From Table 2, first, the proportion of absolute poverty population in contiguous destitute areas decreased significantly in 2019. Among them, the proportion of poverty in contiguous destitute areas of Greater Khingan Range, Liu-P'an Mountains, and Lvliang Mountains is relatively high, and the proportion of poverty in contiguous destitute areas of Greater Khingan Range is relatively high. The proportion of population in Luoxiao Mountains contiguous destitute areas and Ta-pieh Mountains poverty of contiguous destitute areas is relatively small. Second, the proportion of poverty-stricken areas in neighboring southeastern regions is lighter than that in other regions, and the overall poverty situation showed a gradually worsening trend from southeast to northwest. Third, the recognition degree of deep poverty population in various contiguous destitute areas increases with the increase of the deep poverty standard, which is calculated based on the median per capital income of rural residents. In contiguous destitute areas, the proportion of deep poverty population and the poverty situation have been greatly reduced, but the median per capital income of urban residents is taken as the standard. The poverty alleviation of deep poverty population is not stable enough, and rural revitalization is still needed to guide economic development and slow down the deep poverty level in this area.

## 5. Structural Decomposition

### 5.1. Dynamic Change of Poverty Decomposition

According to the change of urban and rural structure and the availability of data, the selection ranges from 2005 to 2011, 2012 to 2015, and 2016 to 2019. Before 2012, it is the stage of rapid urbanization, when the surplus rural labor force is allowed to flow freely; after 2012, it is the stage of rapid urbanization but slowing down. In 2014, it entered a new stage with “people-oriented urbanization” as the core [17]. Furthermore, the structural decomposition of urban and rural poverty changes during this period is performed according to the criteria of deep poverty line and absolute poverty line, respectively.

From Table 3, it can be seen from the results of the change of poverty in 2005–2011 that, first, during 2005–2011, the total effects of economic growth, inequality, and population show the same direction as the change of poverty. Under the criteria of deep poverty line and absolute poverty line, economic growth and population change have a positive effect on the decline of the poverty index of this area, while inequality has a negative effect on the decline of the poverty index. Compared with deep poverty, the effects of these three changes on absolute poverty are not significant enough; second, economic growth, inequality, and population change have different effects on the decline of poverty index, and population change has the strongest effect; third, the higher the poverty line standard, the stronger the positive effect of economic growth on the decline of the poverty index, and the weaker the effect of inequality and population change; fourth, economic growth, inequality, and population change have a stronger impact on the decline of deep poverty index during this period. From the perspective of the corresponding poverty index, absolute poverty has been greatly alleviated, while deep poverty is in a relatively deep state. Economic growth and population change have a positive effect on the large deep poverty group, while inequality has a negative effect.

The decomposition results of poverty change during 2012–2015 can be seen as follows: first, the effect of economic growth on poverty index is opposite to that of inequality and population change. During this period, economic growth has a small impact on poverty index, while inequality and population change have a significant impact on poverty index, which exceeds that of economic growth. The sum of the total effects of the three factors plays a positive role in the decline of the poverty gap index. Second, the higher the poverty line standard, the weaker the influence of economic growth, inequality, and population change on the poverty index. Third, the positive effect of population change is more significant for deep poverty during this period. Compared with the poverty index, the decline rate of deep poverty index is faster than that of absolute poverty index.

It can be seen from the decomposition results of poverty change from 2016 to 2019 that, first, economic growth and inequality have a weak influence on the poverty index, while population change has a strong positive effect on the decline of poverty index. Second, compared with 2012–2015, the influence of population change on deep poverty index is weakened, while the influence on absolute poverty index remained unchanged. Compared with poverty index, during this period, the decline rate of deep poverty index slowed down from the previous month, while the absolute poverty index remained unchanged. Third, the positive effect of inequality at this stage offsets the negative effect of economic growth. Driven by the positive effect of population change, the poverty index showed a downward trend, but the effect of population change weakened, leading to a slower decline of the poverty index.

Changes in poverty in the above three periods can be seen as follows: first, when the degree of deep poverty and absolute poverty is relatively deep, economic growth, inequality, and population change need to play a role together. However, when the degree of poverty slows down, the impact of economic growth and inequality is gradually weakened, while the impact of population change is gradually significant. When the income is at a lower stage, accelerating economic development and narrowing the gap between rich and poor, the reasonable control of natural population growth rate can effectively reduce the extent of poverty, but when the overall income level increased significantly after poverty reduction effect of demographic factors began to be highlighted, on the one hand, population growth period of demographic dividend begins to release and, on the other hand, the household registration system reforms and stimulates labor mobility, which together accelerate the reduction of poverty [20]. Second, when income is at a relatively low stage, the change of inequality has a negative effect on the poverty index. When income increases and the degree of poverty decreases, the change of inequality has a positive effect on poverty reduction. The reason is that when the income level is at a lower stage, the gap between the urban and rural rich and poor is large. At this time, economic growth is in a state of trickle-down growth and urban development is fast, but it is not conducive to poverty reduction in rural areas. After 2013, urban-rural integration began to be implemented, the gap between urban and rural areas gradually narrowed, and economic growth tended to benefit the poor. At this time, the change of inequality played a positive role in poverty reduction. Third, after the income level increases and the degree of poverty is reduced, the effect of population change on poverty reduction decreases, indicating that, after the “flood irrigation” type of poverty alleviation, the degree of poverty is greatly reduced, but the poverty group is gradually solidified and the stickiness of poverty is increased. The poverty reduction effects of economic growth, inequality, and demographic change rarely radiate through this group [21–24].

### 5.2. Intragroup and Intergroup Effect Decomposition

It can be seen from the structural decomposition of poverty change from 2005 to 2019 (Table 4) that, in the process of poverty change, the utility and positive and negative effects of economic growth and inequality change are constantly changing. Only population change has a significant positive effect on the change of poverty. Next, the intergroup effect and intragroup effect are used to conduct the decomposition according to population changes.

According to the results of intragroup effect and intergroup effect decomposition from 2005 to 2011, it can be seen that, first, under different poverty line standards, population change has a positive effect on the decline of the poverty index. From the perspective of the intragroup effect, the slow growth of the total population weakens the dilution effect on the total income, which is conducive to enhancing the positive effect of economic growth within the group and weakening the negative effect of inequality. From the perspective of intergroup effect, population mobility only has an impact on the cities and towns calculated according to the standard of deep poverty line, and the scale of population mobility in this period is limited, which has an insignificant impact on the decline of poverty index. Second, the influence of population change on deep poverty is better than that on absolute poverty. From the perspective of intragroup effect, the dilution effect of total population on total income is more significant in absolute poverty, so the driving effect of economic growth is relatively weak. During this period, the rural population with a relatively high proportion of absolute poverty group is limited by the household registration system, with limited mobility, rapid population growth, and relatively slow economic growth.

From the results of intragroup effect and intergroup effect decomposition during 2012–2015, it can be seen that, first, the effect of population change on the poverty index is significantly enhanced. From the perspective of the intragroup effect, the growth rate of population slows down, the growth rate of poverty population slows down, and the distribution effect of total income on the total population is enhanced, which effectively alleviates the negative effect of inequality. During this period, with the reform of household registration system, the country encouraged the transfer of labor force to cities and allowed rural population to settle in cities. From the perspective of intergroup effect, the positive effect of population mobility on the decline of poverty index is significantly enhanced. Second, the flow of population from rural to urban areas during this period promoted the development of urban areas. Therefore, the intergroup effect and the intragroup effect of population change effectively alleviate deep poverty, and the decline of deep poverty index is also rapid.

From the results of intragroup effect and intergroup effect decomposition during 2016–2019, it can be seen that, first, population change and population movement are the main factors for the decline of poverty index during this period. The intragroup effect of population change is somewhat weakened compared with the first stage, while the intergroup effect of population movement is somewhat enhanced. This indicates that the influence of population change on the change of deep poverty and absolute poverty is gradually weakened, while the influence of population flow on the change of poverty is gradually enhanced. Second, the intergroup effect of population mobility has a significantly better influence on absolute poverty than the previous stage, indicating that rural revitalization promotes rural development, also weakens the flow of rural population to cities and towns, and accelerates the decline of rural poverty index. It effectively drove the remaining absolute poverty units out of poverty.

## 6. Conclusions and Suggestions

### 6.1. Conclusions

FGT index is used to calculate the nature of poverty and structural decomposition of contiguous destitute areas from 2005 to 2019, and to analyze the influence of economic growth, inequality, and population change on the change of poverty. The following main conclusions can be drawn:

  First, poverty of contiguous destitute areas turned to deep poverty. After long-term poverty management, China's poverty index has kept a downward trend, and the proportion of absolute poverty population has been greatly reduced in 2011, and the proportion of deep poverty population has been greatly reduced in 2017. On the whole, absolute poverty has been greatly alleviated, but deep poverty is deeper, and it is more difficult to get rid of poverty. However, deep poverty index keeps a steady downward trend, which indicates that, under the promotion of current policy support and rural revitalization, deep poverty can be effectively managed. The higher the standard of the poverty line, the smaller the range of the poverty index, the weaker the fluctuation of the index, and the longer the time it takes to reduce the degree of poverty. For this reason, after the reduction of deep poverty, efforts should be made to support the contiguous destitute areas in order to get out of the poverty range defined by the high standard deep poverty line.
  Second, the poverty situation showed a trend of aggravation from southeast to northwest. In the contiguous destitute areas, the poverty index of the southeastern provinces is obviously better than that of other areas, mainly because the southeastern provinces have relatively superior economic development conditions, abundant financial support, and rich governance experience in poverty governance. In addition, these subareas are relatively close to large cities, with obvious regional advantages in capital and industrial support, and relatively superior in resource endowment and natural environment. Therefore, the governance effect of poverty is relatively significant. The fragmented areas near the northwest have poor resource endowment with relatively inferior geographical conditions, large difficulty in poverty management, and poor poverty alleviation stability. Therefore, more resources should be allocated to these areas in terms of poverty alleviation stability [25–27].
  Third, population factors have the strongest influence on the whole process of poverty change. The poverty index is positive, which means when the degree of poverty is relatively deep, economic growth and population change are the key factors to promote the change of poverty. Accelerating the economic development in the poverty areas and rationally controlling the population can help the poverty units to get out of poverty. However, the inequality at this stage offsets part of the impact of economic growth and population change on the change of poverty. The reason is that the economy is in a state of trickle-down growth, and poverty units benefit less from economic development by income distribution. Meanwhile, the new population will dilute the total income. The poverty index is negative, which means when the degree of poverty is greatly reduced, inequality and population change are the key factors driving the change of poverty. Promoting labor mobility, controlling population growth, improving the gap between the rich and the poor, and increasing the tilt of primary distribution and redistribution to rural areas are conducive to reducing the degree of poverty. At this stage, the role of population factors is relatively significant, and the influence of inequality on the change of poverty is also gradually significant. The reason is that surplus labor can move freely between urban and rural areas, and the opportunity of obtaining labor remuneration is increased. Meanwhile, the distribution policy guarantees the equality of labor remuneration.
  Fourth, population mobility plays an important role in the process of poverty reduction. According to the results of the intergroup effect and intragroup effect, the population flow presents a strong stage feature and gradually transits from insignificant to significant. The effect of population mobility is not significant. Due to the household registration system, the transfer of rural population to cities is limited, which has little influence on the change of absolute poverty. In the significant stage of population mobility effect, the surplus rural labor force is allowed to move to cities and towns to settle down. During this period, the urban labor force is supplemented, the rural population decreased, and absolute poverty is alleviated. However, the influence of population mobility on absolute poverty is small. With the proposal of rural revitalization, the scale of rural population transfer to cities slows down, and the impact of population flow on the change of deep poverty decreases, while the impact on the change of absolute poverty increases. Therefore, the decline trend of deep poverty index slows down at this stage, while the absolute poverty index basically remains unchanged.

### 6.2. Suggestions

  First, we should promote the organic link between rural revitalization and poverty alleviation and consolidate the achievements of poverty alleviation through rural revitalization. To grasp the connection between urbanization, industrialization, and rural development from the overall perspective, we need to accelerate rural development and realize the modernization of rural governance. In the process of development, it is firmly focused on the “two without worry and three guarantees” to accurately connect with poverty units, stabilize the income of poverty population, and eliminate absolute poverty. Problems in poverty governance can be included in rural revitalization planning and solved gradually by relying on rural governance.
  Second, we are supposed to improve the employment service system to attract the return of population. Population change is the key factor in the change of poverty, among which population migration has a more significant impact on poverty. Therefore, in rural development and governance, the return of labor is needed. In contiguous destitute areas, it is necessary to improve the macroscopic employment environment and provide employment opportunities according to their own characteristics. Meanwhile, it is necessary to provide skills training for poverty units from two perspectives of “supporting orientation” and “supporting intelligence,” so as to cultivate specialized farmers suitable for local development.
  Third, we need to promote a new type of urbanization and narrow the urban-rural gap. The deep poverty index of different deep poverty standards has great differences, and the degree of deep poverty of contiguous destitute areas is relatively deep when the median per capital income of urban residents is the calculation standard. Therefore, it is necessary to promote the integration of urban and rural areas, improve the quality of urbanization and the rural living environment, perfect the rural infrastructure construction, increase the rural carrying capacity, speed up the return of labor force, alleviate the rural hollowing out problem, and further promote the rural development [28–30].

## Figures and Tables

**Table 1:** Poverty index of contiguous destitute areas index from 2005 to 2019.

Years	Deep poverty line			Absolute poverty line
	40%	50%	60%	
2005	3.243	1.609	1.720	0.766
2006	2.935	1.441	1.528	0.642
2007	2.634	1.279	1.355	0.525
2008	2.372	1.141	1.202	0.397
2009	2.118	1.006	1.051	0.299
2010	1.861	0.867	0.879	0.190
2011	1.608	0.732	0.710	0.045
2012	1.316	0.585	0.549	−0.056
2013	1.030	0.433	0.367	−0.171
2014	0.827	0.318	0.231	−0.259
2015	0.681	0.241	0.161	−0.314
2016	0.471	0.139	0.052	−0.377
2017	0.256	0.037	−0.045	−0.426
2018	0.122	−0.026	−0.103	−0.470
2019	0.009	−0.080	−0.159	−0.516

**Table 2:** Poverty index of contiguous destitute areas in 2019.

Areas	Deep poverty line			Absolute poverty line
	40%	50%	60%	
Ta-pieh Mountains	0.007	−0.054	−0.162	−0.531
Greater Khingan Range	0.008	−0.074	−0.169	−0.573
West Yunnan border mountainous area	0.008	−0.066	−0.116	−0.256
Liu-p'an Mountains	0.013	−0.123	−0.168	−0.570
Luoxiao Mountains	0.007	−0.056	−0.092	−0.112
Lvliang Mountains	0.010	−0.089	−0.152	−0.475
Tsinling Mountains	0.008	−0.073	−0.157	−0.499
Rocky desertification areas in Yunnan, Guangxi, and Guizhou	0.010	−0.087	−0.215	−0.851
Yanshan Mountains, T'ai-hang Mountains	0.010	−0.089	−0.164	−0.545
Wumeng Mountains	0.009	−0.078	−0.155	−0.510
Wuling Mountains	0.009	−0.080	−0.159	−0.516

**Table 3:** Structural decomposition of poverty of contiguous destitute areas from 2005 to 2019.

Years	Effect	Deep poverty line			Absolute poverty line
		40%	50%	60%	
2005–2011	Economic growth	−0.022	−0.106	−0.073	−0.018
	Inequality	−0.044	−0.005	−0.145	−0.001
	Population	−0.075	−0.143	−0.250	−0.024

2012–2015	Economic growth	0.035	0.023	0.137	−0.006
	Inequality	0.031	0.170	0.124	−0.047
	Population	−0.127	−0.288	−0.499	−0.022

2016–2019	Economic growth	0.002	−0.001	0.005	0.000
	Inequality	0.028	0.032	0.073	0.007
	Population	−0.108	−0.136	−0.279	−0.028

**Table 4:** Intragroup and intergroup effect decomposition from 2005 to 2019.

Years	Poverty line standard		Intergroup effect population	Intragroup effect		
				Economic growth	Inequality	Population
2005–2011	Deep poverty	40%	−0.4676	−0.0204	−0.0117	−0.0232
		50%	0.1982	−0.0158	−0.0435	−0.0763
		60%	0.3100	0.0008	−0.0004	−0.0129
	Absolute poverty		0.6291	0.0018	−0.0009	−0.0241

2012–2015	Deep poverty	40%	−0.1755	−0.0030	0.0106	−0.0439
		50%	−0.0604	0.0407	0.0314	−0.1277
		60%	−0.0469	0.0114	0.0142	−0.0249
	Absolute poverty		0.0108	0.0130	−0.0469	−0.0794

2016–2019	Deep poverty	40%	−0.2014	−0.0140	0.0064	−0.0256
		50%	−0.1287	0.0080	0.0284	−0.1090
		60%	−0.0819	0.0094	0.0027	−0.0122
	Absolute poverty		−0.0241	0.0191	0.0065	−0.0283

## Data Availability

The data used to support the findings of this study are included within the article.
